# Stress fracture of the posterior talar process in a female long-distance runner treated by osteosynthesis with screw fixation via two-portal hindfoot endoscopy: a case report

**DOI:** 10.1186/s12891-019-2774-y

**Published:** 2019-09-03

**Authors:** Maya Kubo, Youichi Yasui, Shinya Miki, Hirotaka Kawano, Wataru Miyamoto

**Affiliations:** Department of Orthopaedic Surgery, Teikyo University School of Medicine, 2-11-1 Kaga, Itabashi, Tokyo, 173-8605 Japan

**Keywords:** Stress fracture, Talus, Athlete, Minimal invasive surgery

## Abstract

**Background:**

Stress fracture of the lateral tubercle of the posterior talar process in runners is extremely rare. Here, we describe a case of a female long-distance runner who sustained a stress fracture of the lateral tubercle of the posterior talar process. Osteosynthesis with screw fixation via two-portal hindfoot endoscopy achieved a good surgical outcome with a less invasive procedure.

**Case presentation:**

An 18-year-old female long-distance runner who belonged to her university’s road running club presented to our institution with a half-year history of persistent left hindfoot pain when running. Radiographs revealed a stress fracture of the lateral tubercle of the posterior talar process. Because the fracture showed no signs of healing 3 months after starting conservative therapy, osteosynthesis with screw fixation was performed via two-portal hindfoot endoscopy. Non-contrast computed tomography at 10 weeks postoperatively revealed consolidation of the stress fracture. At 15 weeks postoperatively, the patient was permitted to jog and return to athletic activity while wearing an orthosis. As of this writing 2 years postoperatively, she remains an active competitive runner.

**Conclusions:**

Osteosynthesis with screw fixation via two-portal hindfoot endoscopy was a less invasive procedure that successfully treated stress fracture of the lateral tubercle of the posterior talar process in this female long-distance runner.

## Background

Stress fractures affect approximately 1 to 7% of athletes. Runners have an especially high risk for this skeletal disorder, with the prevalence reaching as high as 20% [1, 2]. In previous reports evaluating 320 athletes with stress fracture, bones of the lower limb were more likely to be affected. The tibia was most commonly involved, followed by the tarsal bones, such as the calcaneus and navicular, and the metatarsals [3]. Stress fracture of the talus seems to be relatively rare, and with a reported incidence of only 4.4 cases per 10,000 person-years in one study of military recruits [4]. In stress fractures of the talus, the talar head is most commonly involved, followed by the talar body. Involvement of the posterior talus is less common [4]. To our knowledge, stress fracture of the lateral tubercle of the posterior talar process in runners has not been reported.

Posterior ankle impingement syndrome (PAIS) is a clinical disorder characterized by posterior ankle pain in forced plantarflexion. Various causes of PAIS have been reported, such as fracture of the trigonal process, os trigonum synchondrosis, tenosynovitis of the flexor hallucis longus, subtalar osteochondritis or arthritis, and calcification of inflammatory tissue [5–7]. However, there has been no report attributing PAIS to stress fracture of the lateral tubercle of the posterior talar process.

Here, we describe a case of a female long-distance runner who sustained a stress fracture of the lateral tubercle of the posterior talar process, leading to PAIS. Because this disorder did not respond to conservative therapy, surgical therapy was applied. Osteosynthesis with screw fixation was performed via two-portal hindfoot endoscopy achieving a good surgical outcome with a less invasive procedure. Written informed consent was obtained from the patient for publishing this case report, including the images.

## Case presentation

An 18-year-old female long-distance runner who belonged to her university’s road running club presented to our institution with a half-year history of persistent left hindfoot pain when running. She was of Japanese ethnicity and her height and weight were 162 cm and 45.1 kg, respectively. Her body mass index was 17.2. She had no history of previous trauma. However, she had a medical history of amenorrhea and iron deficiency anemia, which responded to iron supplementation. Her weekly training regimen consisted of cross-country jogging for 120 km, track jogging for 200 min, and speed exercise for 200 min.

**Fig. 1:**
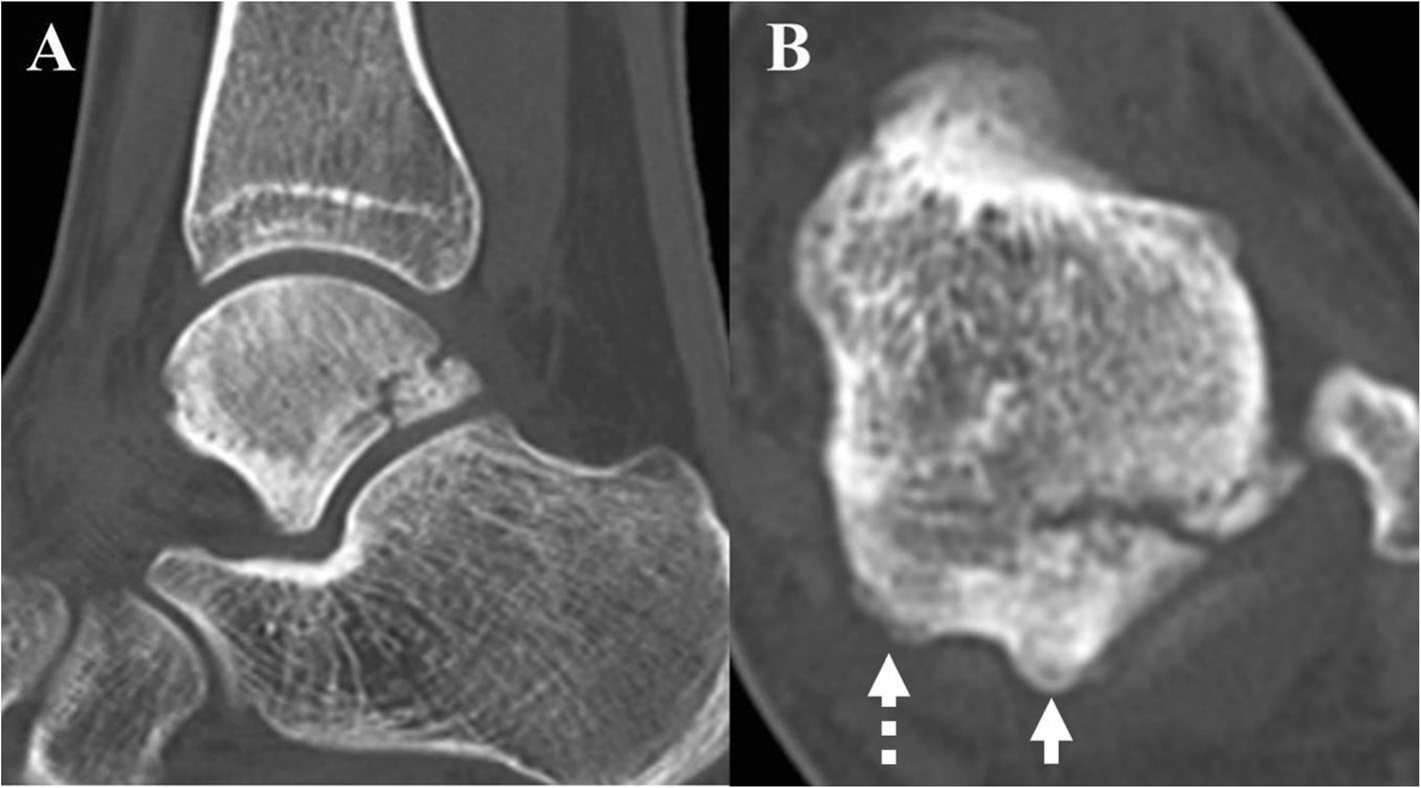
Preoperative non-contrast CT scan. **a** Sagittal and (**b**) axial views showing the fracture line located just lateral to the groove for flexor hallucis longus (FHL) tendon at a level just proximal to the subtalar joint. Posterior process of the talus comprising the medial (dotted arrow) and lateral (arrow) tubercles, separated by the groove for the FHL tendon. CT, computed tomography

Physical examination revealed hindfoot valgus of both feet and swelling on the hindfoot region and tenderness in the area slightly lateral to the lateral border of the Achilles tendon at the level of the distal malleolus. When the left ankle joint was forced into plantarflexion, she complained of pain in the left hindfoot region. However, ankle and hindfoot motion were within the full range. Plain radiographs of the left ankle showed no abnormality. Non-contrast computed tomography (CT) revealed a fracture line in the lateral tubercle of the posterior talar process. The fracture line was located just lateral to the groove for the flexor hallucis longus (FHL) tendon in the axial plane and positioned just proximal to the subtalar joint in the sagittal plane (Fig. 1). Dual-energy X-ray absorption revealed that the Z-score was less than − 1.0, which was diagnosed as low bone density based on the diagnostic criteria of the American College of Sports Medicine [8]. Based on the physical examination, medical history, and radiological findings, the diagnosis was stress fracture in the lateral tubercle of the posterior talar process. Immediately after diagnosis, conservative therapy, including non-weightbearing using a patellar tendon-bearing brace and low-intensity pulsed ultrasound, was started to facilitate consolidation of the fracture. However, the fracture did not show any signs of healing 3 months after instituting conservative therapy, so we opted for surgical intervention via two-portal access hindfoot endoscopy.

**Fig. 2:**
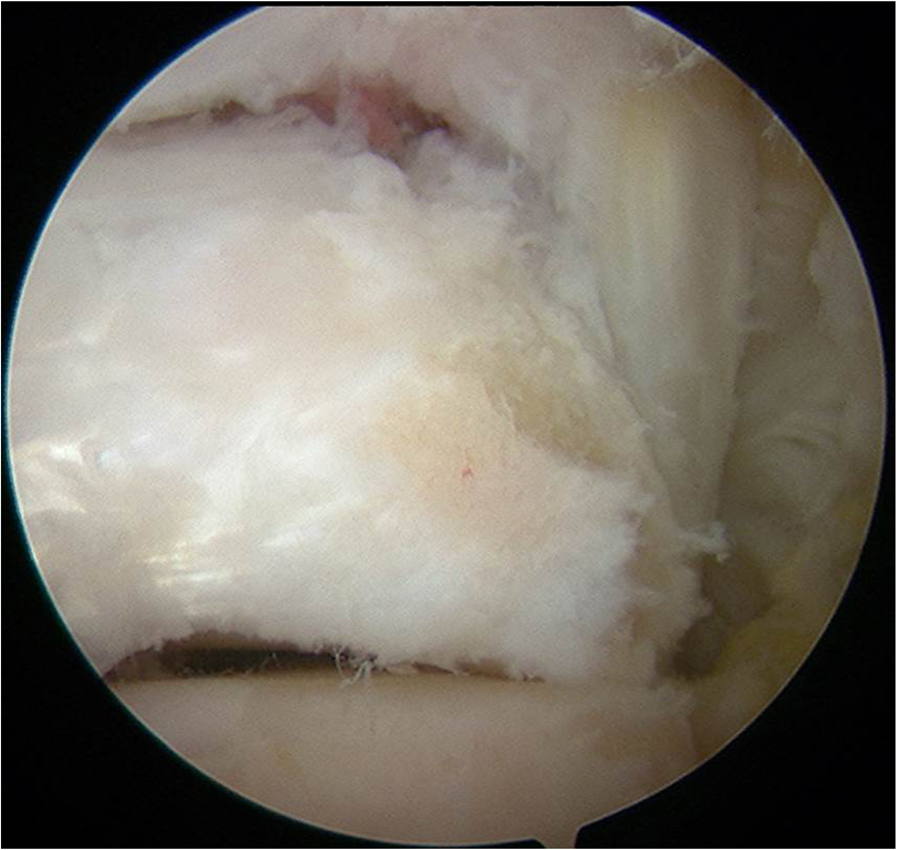
Endoscopic view of landmarks for screw insertion. The subtalar joint and FHL tendon are shown, which were landmarks for screw insertion

**Fig. 3:**
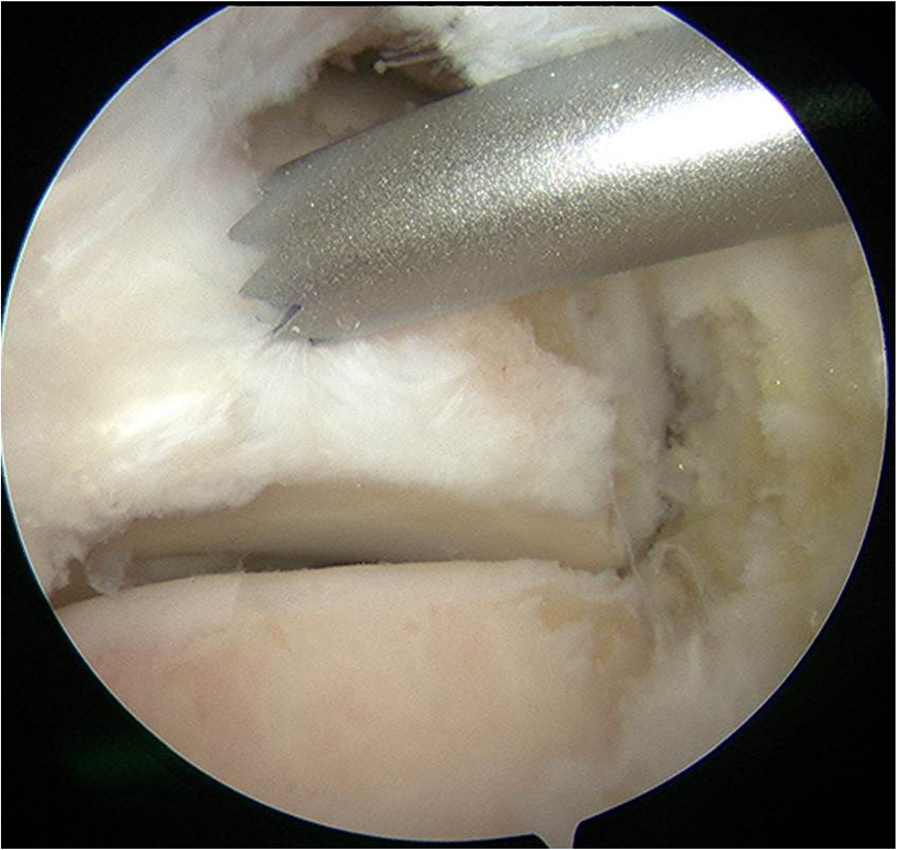
Endoscopic view of guidewire insertion. The guidewire was inserted approximately 5 mm lateral to the FHL tendon just above the subtalar joint level

**Fig. 4:**
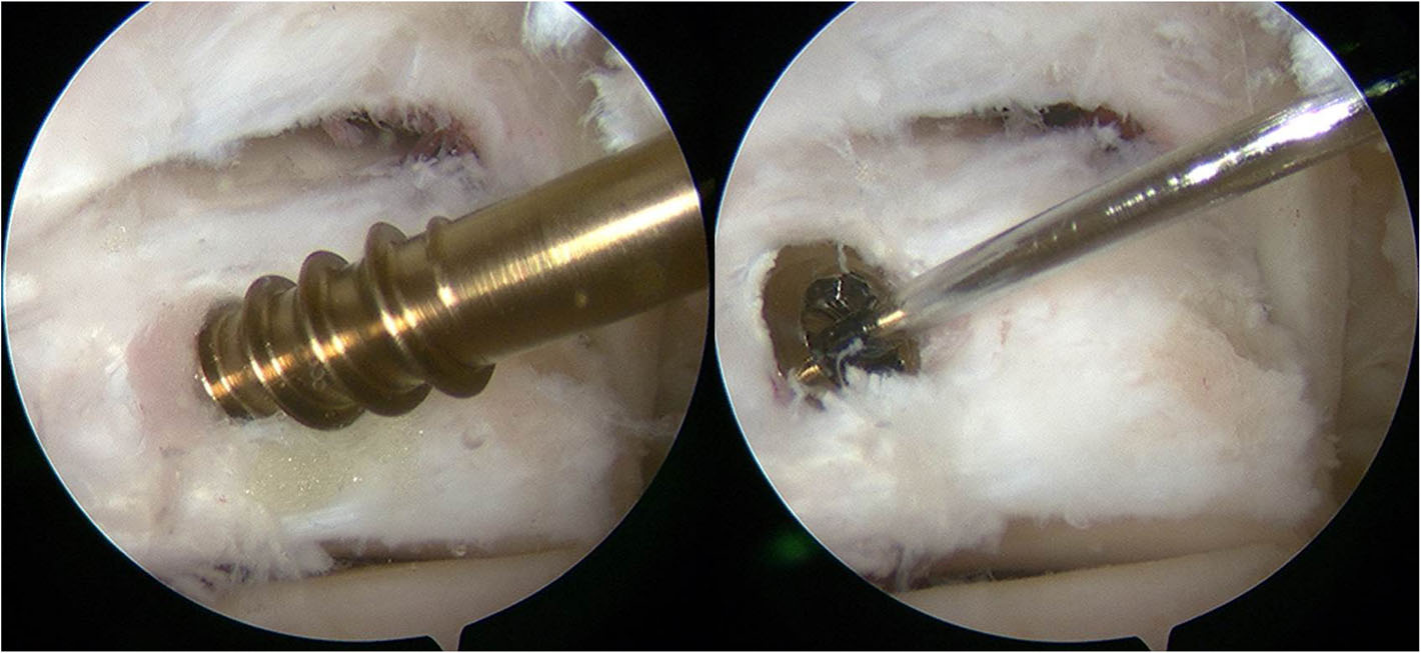
Endoscopic view of screw insertion. A cannulated double-thread screw (diameter, 3 mm) was inserted through the guidewire to fix the fracture

The operation was performed using a pneumatic tourniquet (280 mmHg) and general anesthesia with the patient in the prone position. Based on the procedure reported by van Dijk et al., posterolateral and posteromedial portals were created at the level of the tip of the lateral malleolus, just medial and lateral to the Achilles tendon [9]. A 4.0-mm arthroscope with a 30° angle was introduced through the posterolateral portal, while a 4.0-mm motorized shaver was introduced through the posteromedial portal. Adipose tissue was dissected with the motorized shaver exposing the subtalar joint and the FHL tendon, which were landmarks for the insertion point of the screw (Fig. 2). These landmarks were exposed under endoscopy, and a guidewire was then inserted through a sleeve approximately 5 mm lateral to the FHL tendon at a level just above the subtalar joint (Fig. 3) through the posteromedial portal. This seemed to be an adequate position to penetrate the fracture line according to preoperative radiological evaluation. Through the guidewire, a cannulated 3-mm double-threaded screw was inserted to fix the fracture in the lateral tubercle of the posterior talar process (Fig. 4). After irrigation of the hindfoot space, each portal was sutured.

**Fig. 5:**
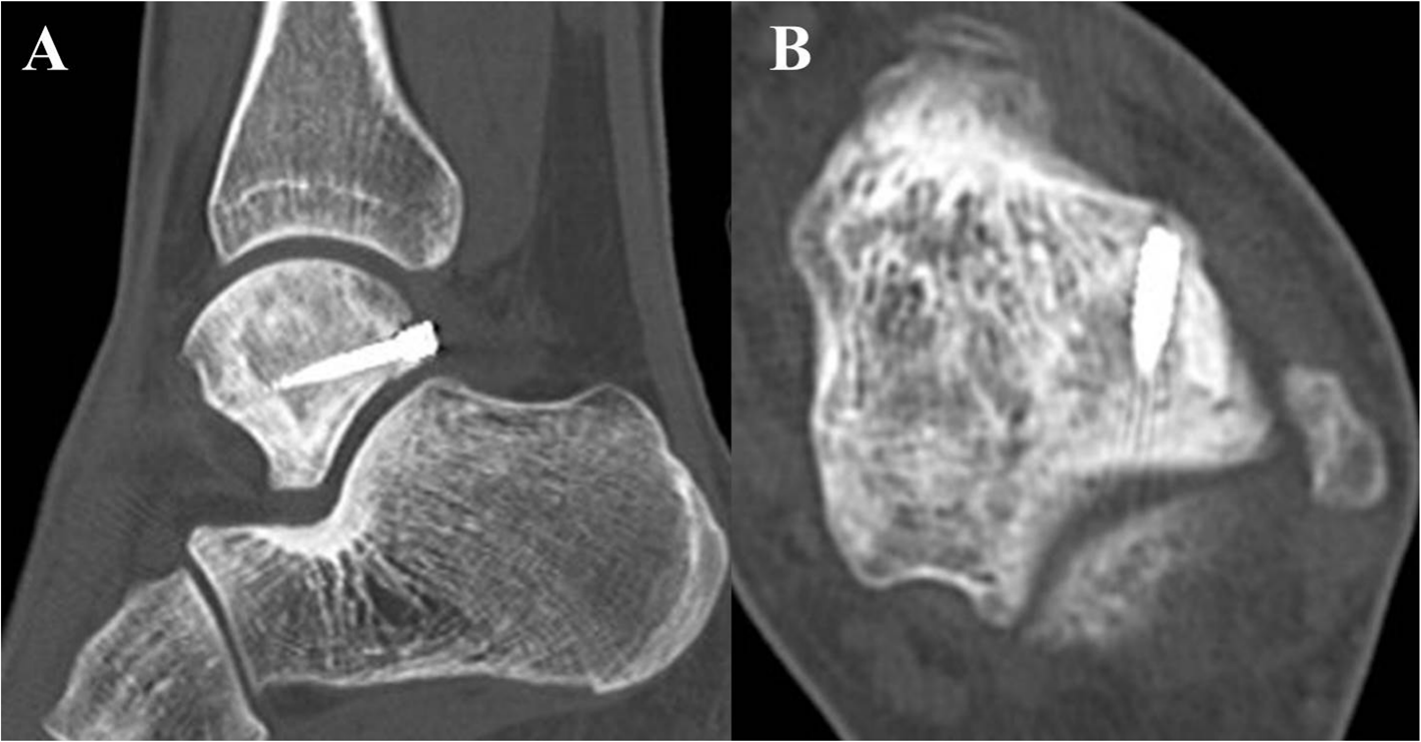
Postoperative non-contrast CT scan. **a** Sagittal and **b** axial views at 10 weeks postoperatively showing consolidation of the stress fracture

Postoperatively, a short leg cast was applied for 4 weeks, and then walking using a patellar tendon-bearing brace was started. Active range of motion exercises were allowed 4 weeks postoperatively, and non-weightbearing was continued until 10 weeks postoperatively. Non-contrast CT at 10 weeks postoperatively revealed consolidation of the stress fracture (Fig. 5), so the brace was removed and full weightbearing was started. At 15 weeks postoperatively, the patient was permitted to jog and return to athletic activity while wearing an orthosis. At that time, her running form was examined and she was found to have overpronation at heel strike when running without the orthosis. Her running coach and trainer worked with her to improve her running form, core strength, and static and dynamic alignment. She had no relevant symptoms 5 months postoperatively. As of this writing 2 years postoperative, she remains an active competitive runner.

## Discussion

There has been no previous report of stress fracture of the lateral tubercle of the posterior talar process causing PAIS. Stress fracture has historically been divided into high- and low-risk injuries [10, 11]. Although most stress fractures of the foot are low risk, talar stress fractures are high-risk injuries associated with delayed healing, leading to chronic ankle pain and even necrosis [4, 12]. In the present case, the patient had a medical history of amenorrhea and iron deficiency anemia due to chronic low energy availability [13]. There is a widespread hypothesis that prolonged low energy availability in female runners causes depression of the cyclic secretion of luteinizing hormone from the pituitary gland leading to amenorrhea [13]. Furthermore, amenorrhea reduces secretion of estrogen from the ovaries resulting in low bone density. Low energy availability has been reported to be a risk factor for stress fracture in female runners, increasing the risk by 2.4 to 6.8-fold [14]. In addition, the mechanism underlying this stress fracture is considered to be the presence of both excessive subtalar pronation and repetitive forced plantarflexion in running, causing inevitable impingement between the lateral process of the calcaneus and the posterolateral corner of the talus [15, 16].

The literature on talar stress fracture is sparse. To our knowledge, there have been only 4 reports, some of which included multiple cases [4, 16–18]. The largest series reported 5 cases with stress fracture of the posterior aspect of the talus [4]. In all 5 cases, there was no fracture line on plain radiographs but endosteal edema of the talus on magnetic resonance imaging (MRI); our case also showed no abnormality on plain radiographs but a fracture line on CT. Similar to other stress fractures [19, 20], this difference suggests that stress fracture of the posterior aspect of the talus is a sequence progressing from microdamage through 4 stages up to significant fracture of the talus. Also, the similarity between previous cases and ours suggests a noteworthy diagnostic algorithm where additional radiological examinations such as MRI or CT should be performed in cases of persistent ankle pain even if no fracture line is detected on plain radiographs.

Surgical outcomes of acute posterior talar process fractures or nonunion have been described in several previous reports, all of which applied osteosynthesis with screw fixation through an open posteromedial approach and achieved good outcomes [21–25]. However, osteosynthesis through an open posteromedial approach requires a large skin incision because the lateral tubercle of the posterior talar process is located deep in the hindfoot where the anatomic structures are in close proximity to each other. Such an invasive procedure may delay return to athletic activity for long-distance runners. In the present case, osteosynthesis with screw fixation was performed via two-portal hindfoot endoscopy in order to use a less invasive approach. This allowed the patient to make an early return to athletic activity. Preoperative non-contrast CT scan revealed a fracture line located just lateral to the groove for FHL tendon in the axial plane and positioned at a level just proximal to the subtalar joint in the sagittal plane. These detailed findings on preoperative CT scan suggest the subtalar joint and the FHL tendon as effective landmarks for hindfoot endoscopic screw insertion. After identifying the subtalar joint and the FHL tendon using hindfoot endoscopy, the screw was inserted just lateral to the FHL tendon at a level just proximal to the subtalar joint. This procedure could serve to avoid injury to the neurovascular structures positioned just medial to the FHL tendon.

In female runners with a history of amenorrhea and anemia who have foot and ankle symptoms without a history of trauma, surgeons should consider stress fracture as a differential diagnosis even when no fracture line is detected on plain radiographs. Stress fracture in the lateral tubercle of the posterior talar process is rare but can occur, potentially leading to PAIS. Osteosynthesis with screw fixation via hindfoot endoscopy is a less invasive surgery for stress fracture in the lateral tubercle of the posterior talar process. This procedure was effective for fixing the fracture securely without injury to the neurovascular structures and allowing an early return to athletic activity.

## Conclusion

We described a case of a female long-distance runner who sustained stress fracture of the lateral tubercle of the posterior talar process. In this case, the stress fracture resulted in PAIS, which is a clinical disorder characterized by posterior ankle pain in forced plantarflexion. Osteosynthesis with screw fixation via two-portal hindfoot endoscopy was used a less invasive surgery for stress fracture in the lateral tubercle of the posterior talar process. This procedure avoided open surgery and was effective for consolidation of the fracture and an early return to athletic activity.

## Data Availability

The datasets analyzed in this study are available from the corresponding author on reasonable request.

## Abbreviations

CT: Computed tomography
FHL: Flexor hallucis longus
MRI: Magnetic resonance imaging
PAIS: Posterior ankle impingement syndrome

## References

[1] Boden BP, Osbahr DC (2000). High-risk stress fractures: evaluation and treatment. J Am Acad Orthop Surg..

[2] Gehrmann RM, Renard RL (2006). Current concepts review: stress fractures of the foot. Foot Ankle Int..

[3] Matheson GO, Clement DB, McKenzie DC, Taunton JE, Lloyd-Smith DR, MacIntyre JG (1987). Stress fractures in athletes. A study of 320 cases. Am J Sports Med.

[4] Sormaala MJ, Niva MH, Kiuru MJ, Mattila VM, Pihlajamäki HK (2006). Bone stress injuries of the talus in military recruits. Bone.

[5] Hsu AR, Gross CE, Lee S, Carreira DS (2014). Extended indications for foot and ankle arthroscopy. J Am Acad Orthop Surg.

[6] Maquirriain J (2005). Posterior ankle impingement syndrome. J Am Acad Orthop Surg.

[7] van Dijk CN, van Bergen CJ (2008). Advancements in ankle arthroscopy. J Am Acad Orthop Surg.

[8] De Souza MJ, Nattiv A, Joy E, Misra M, Williams NI, Mallinson RJ, et al. 2014 Female athlete triad coalition consensus statement on treatment and return to play of the female athlete triad: 1st international conference held in San Francisco, California, may 2012 and 2nd international conference held in Indianapolis, Indiana, may 2013. Br J Sports Med. 2014;48:289. 10.1136/bjsports-2013-093218 24463911

[9] van Dijk CN, Scholten PE, Krips R (2000). A 2-portal endoscopic approach for diagnosis and treatment of posterior ankle pathology. Arthroscopy.

[10] Boden BP, Osbahr DC (2000). High-risk stress fractures: evaluation and treatment. J Am Acad Orthop Surg.

[11] Boden BP, Osbahr DC, Jimenez C (2001). Low-risk stress fractures. Am J Sports Med.

[12] Travlos J, Learmonth ID (1991). Bilateral avascular necrosis of the talus following strenuous physical activity. J Bone Joint Surg Br.

[13] Loucks AB, Thuma JR (2003). Luteinizing hormone pulsatility is disrupted at a threshold of energy availability in regularly menstruating women. J Clin Endocrinol Metab J.

[14] Mallinson RJ, De Souza MJ (2014). Current perspectives on the etiology and manifestation of the “silent” component of the female athlete triad. Int J Women's Health.

[15] Kou JX, Fortin PT (2009). Commonly missed peritalar injuries. J Am Acad Orthop Surg.

[16] Bradshaw C, Khan K, Brukner P (1996). Stress fracture of the body of the talus in athletes demonstrated with computer tomography. Clin J Sport Med.

[17] Gilbert RS, Crawford AH, Rankin E (1980). Stress fractures of the tarsal talus. Sports Med.

[18] Rossi F, Dragoni S (2005). Talar body fatigue stress fractures: three cases observed in elite female gymnasts. Skelet Radiol.

[19] Kiuru MJ, Pihlajamäki HK, Perkiö JP, Ahovuo JA (2001). Dynamic contrast-enhanced MR imaging in symptomatic bone stress of the pelvis and the lower extremity. Acta Radiol.

[20] Kiuru MJ, Niva MH, Reponen A, Pihlajamaki HK (2005). Bone stress injuries in asymptomatic elite recruits. A clinical and MRI study. Am J Sports Med.

[21] Bhanot A, Kaushal R, Bhan R, Gupta PN, Gupta RK, Bahadur R (2004). Fracture of the posterior process of talus. Injury.

[22] Dougall TW, Ashcroft GP (1997). Flexor hallucis longus tendon interposition in a fracture of the medial tubercle of the posterior process of the talus. Injury.

[23] Hsu AR, Scolaro JA (2016). Posteromedial approach for open reduction and internal fixation of talar process fractures. Foot Ankle Int.

[24] Nadim Y, Tosic A, Ebraheim N (1999). Open reduction and internal fixation of fracture of the posterior process of the talus: a case report and review of the literature. Foot Ankle Int.

[25] Nakai T, Murao R, Temporin K, Kakiuchi M (2005). Painful nonunion of fracture of the entire posterior process of the talus: a case report. Arch Orthop Trauma Surg.

